# Supplementary material for: Exploring the Value of BRD9 as a Biomarker, Therapeutic Target and Co-Target in Prostate Cancer
Source: Biomolecules. 2021 Nov 30;11(12):1794. doi: 10.3390/biom11121794 (PMC8698755; doi:10.3390/biom11121794)
Supplement: Supplementary file 1 [file biomolecules-11-01794-s001.zip › biomolecules-1387975 - supplementary.pdf]

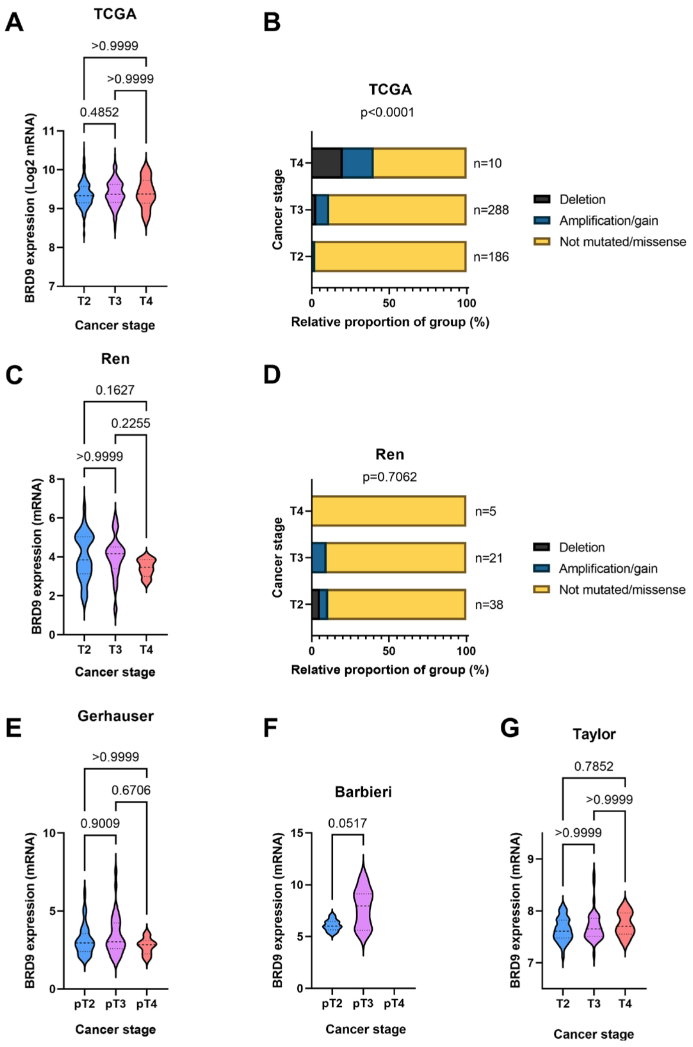

Figure S1. *BRD9* expression by cancer stage in cancer omics cohorts. (**A**) Violin plot showing how *BRD9* expression varies with cancer stage in the TCGA cohort. P-values were obtained using Dunn's post-hoc test. (**B**) Stacked bar chart showing *BRD9* mutation distribution at different cancer stages and the number of patients at each stage in the TCGA cohort. P-value was obtained using Fisher's exact test. (**C**) Violin plot showing how *BRD9* expression varies with cancer stage in the Ren cohort. P-values were obtained using Dunnett's T3 post-hoc test. (**D**) Stacked bar chart showing *BRD9* mutation distribution at different cancer stages and the number of patients at each stage in the Ren cohort. P-value was obtained using Fisher's exact test. (**E**) Violin plot showing how *BRD9* expression varies with cancer stage in the Gerhauser cohort. P-values were obtained using Dunn's post-hoc test. (**F**) Violin plot showing how *BRD9* expression varies with cancer stage in the Barbieri cohort. P-values were obtained using Dunnett's T3 post-hoc test. (**G**) Violin plot showing how *BRD9* expression varies with cancer stage in the Taylor cohort. P-values were obtained using Dunn's T3 post-hoc test. Stages were combined into three stage groups due to small sample sizes (n) at individual stages (e.g. T2a). Y axis scales on violin plots vary due to experimental variation.

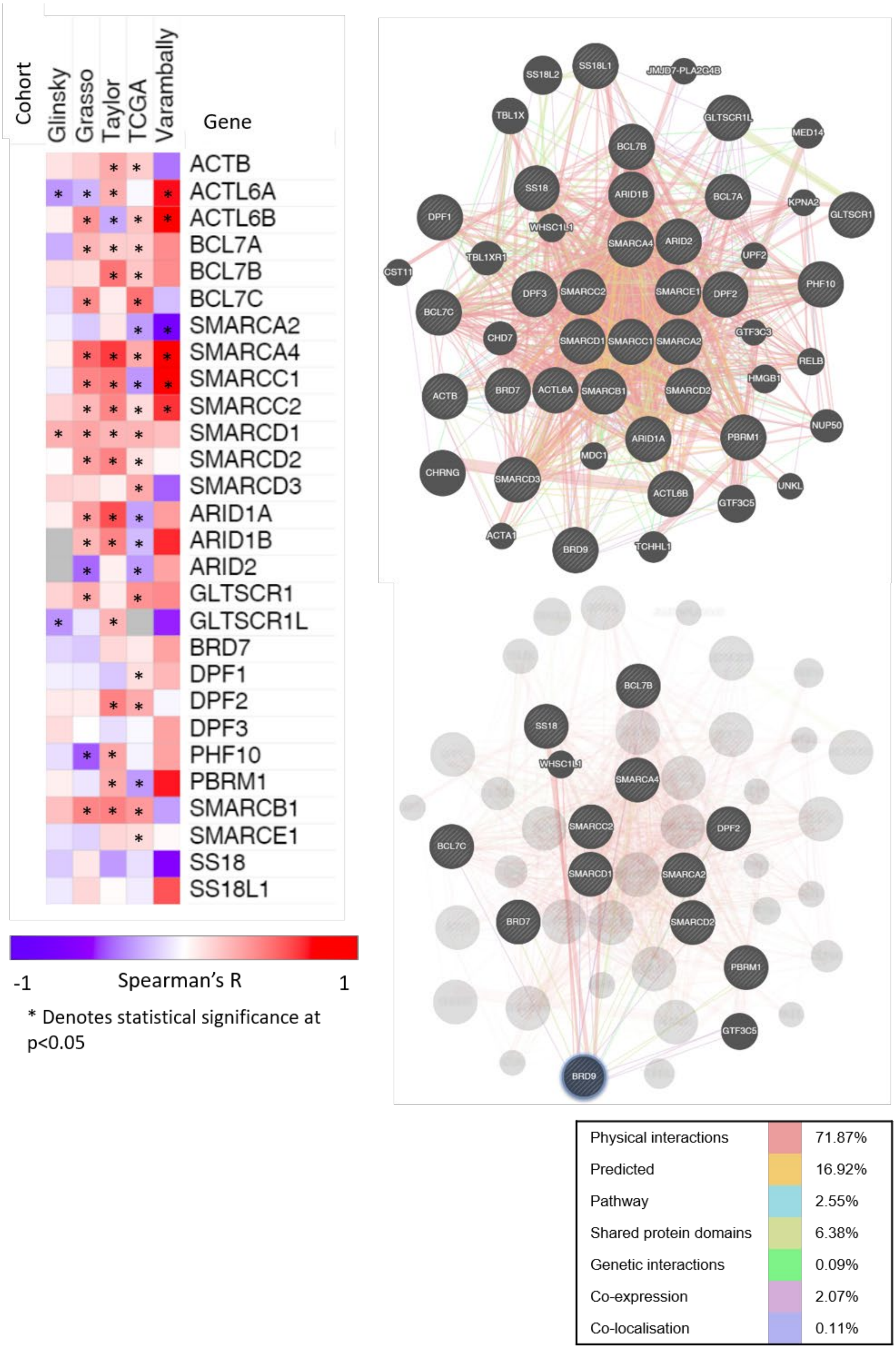

Figure S2. Correlation and association of *BRD9* with genes composing the SWI/SNF complex. (**A**) Heatmap showing the correlation (Spearman's R coefficient) between *BRD9* and genes that make up the SWI/SNF complex. Statistically significant correlations (p<0.05) are indicated by '*'. (**B**) Gene

network map showing the associations between *BRD9* and known genes that make up the SWI/SNF complex. Heatmap made using https://software.broadinstitute.org/morpheus/ with statistically significant correlation denotation added in Microsoft PowerPoint. Gene network map created using https://genemania.org/.

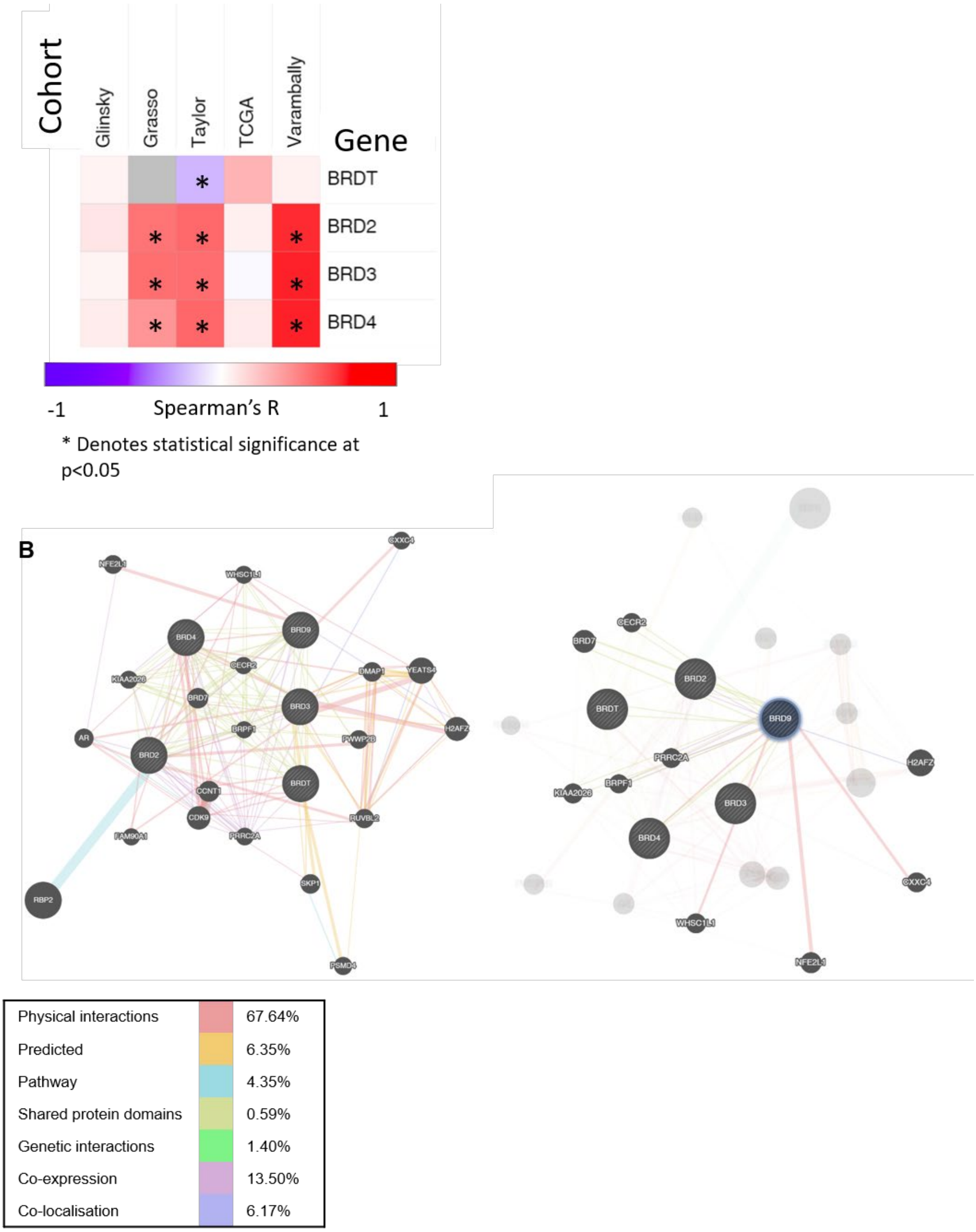

Figure S3. Correlation and association of *BRD9* with genes composing the BET complex. (**A**) Heatmap showing the correlation (Spearman's R coefficient) between *BRD9* and genes that make up the BET complex. Statistically significant correlations (p<0.05) are indicated by '*'. (**B**) Gene network map showing the associations between *BRD9* and known genes that make up the BET complex. Heatmap made using https://software.broadinstitute.org/morpheus/ with statistically significant

correlation denotation added in Microsoft PowerPoint. Gene network map created using https://genemania.org/.

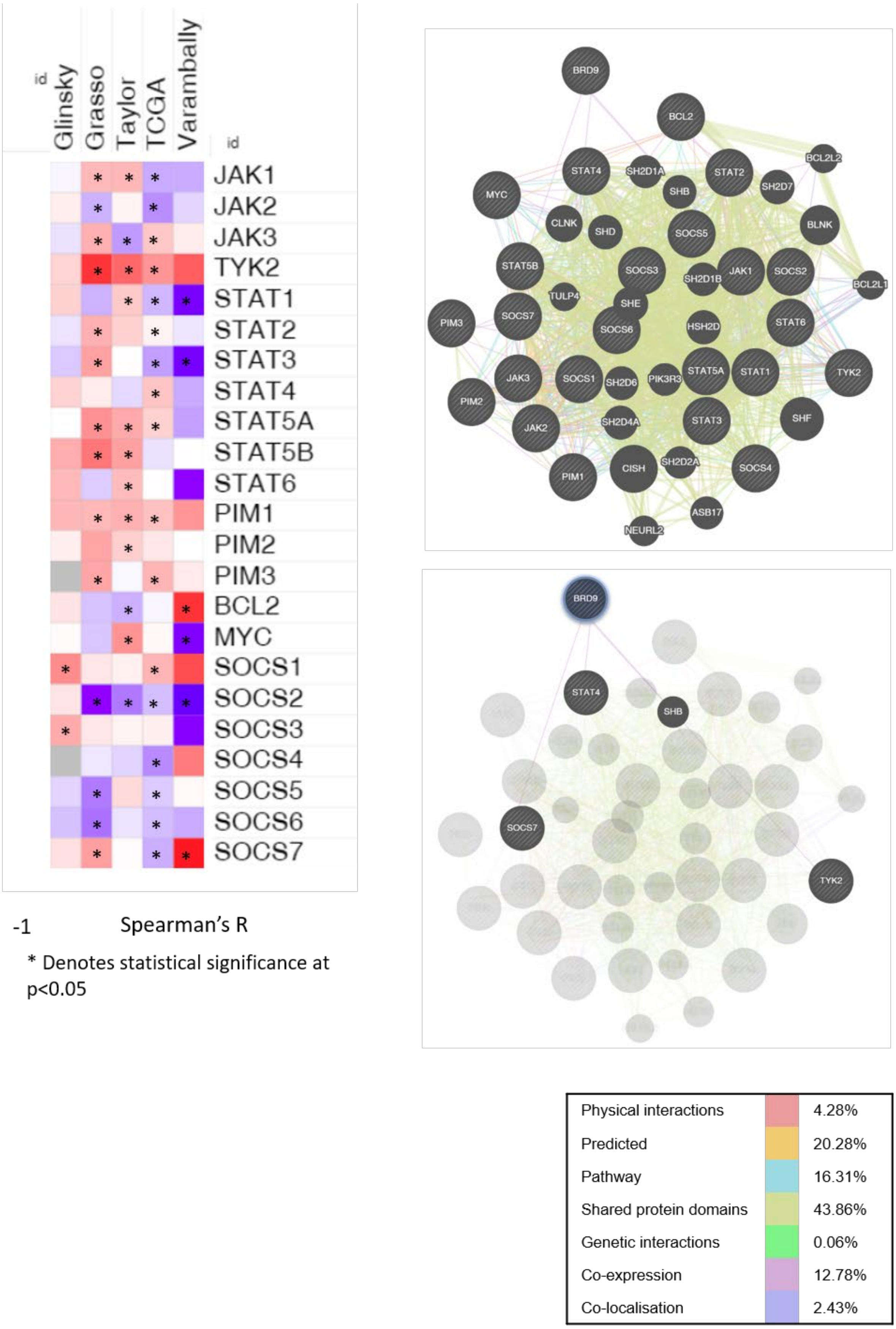

Figure S4. The correlation and association of *BRD9* with genes expressed in the JAK-STAT pathway. (**A**) Heatmap showing the correlation (Spearman's R coefficient) between *BRD9* and genes involved in the JAK-STAT pathway. Statistically significant correlations ($p<0.05$) are indicated by '*'. (**B**) Gene network map showing the associations between *BRD9* and known genes expressed in the JAK-STAT

pathway. Heatmap made using https://software.broadinstitute.org/morpheus/ with statistically significant correlation denotation added in Microsoft PowerPoint. Gene network map created using https://genemania.org/.

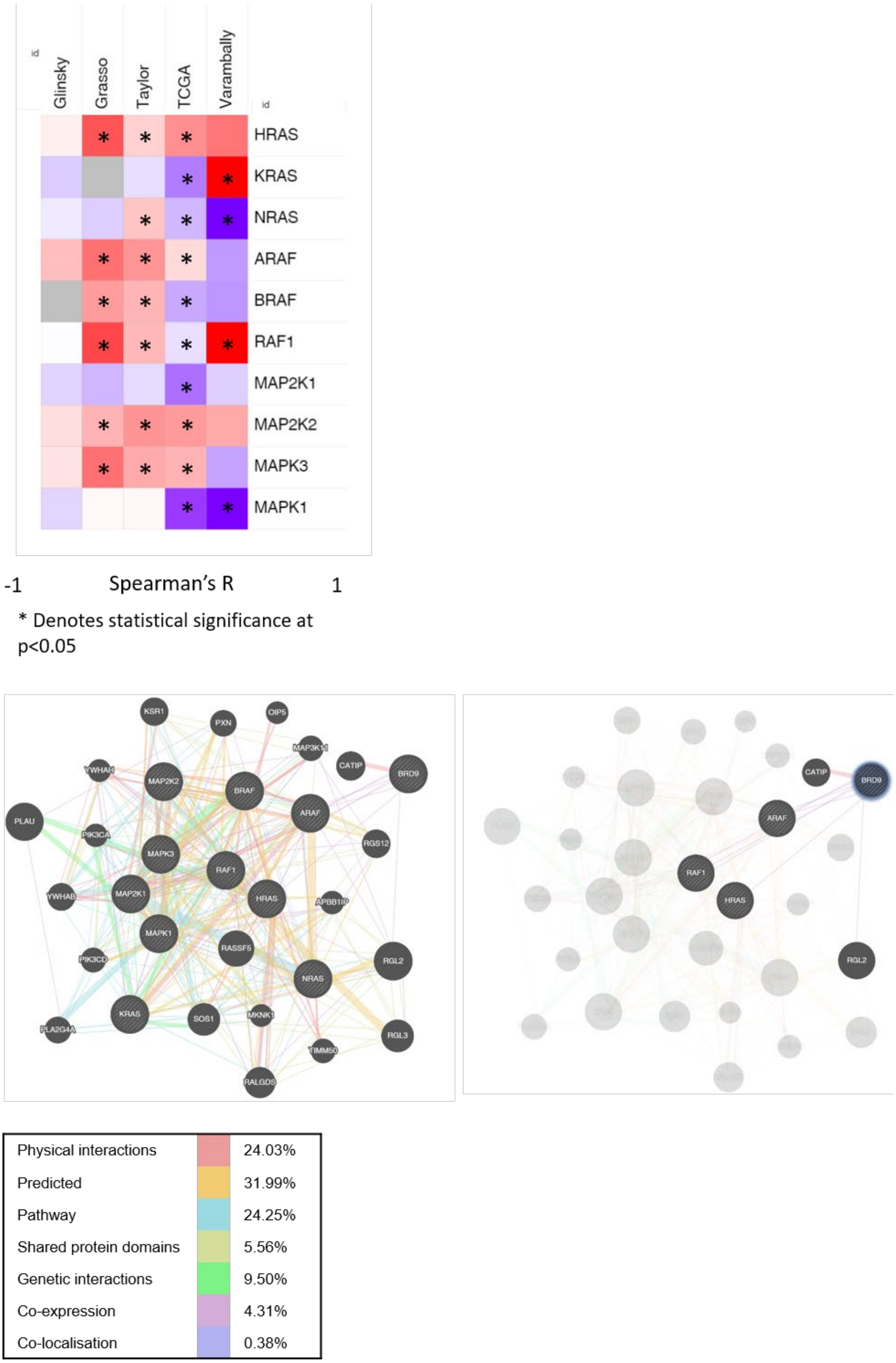

Figure S5. The correlation and association of *BRD9* with genes expressed in the MAPK pathway. (**A**) Heatmap showing the correlation (Spearman's R coefficient) between *BRD9* and genes involved in the MAPK pathway. Statistically significant correlations ($p<0.05$) are indicated by '*'. (**B**) Gene

network map showing the associations between *BRD9* and known genes expressed in the MAPK pathway. Heatmap made using https://software.broadinstitute.org/morpheus/ with statistically significant correlation denotation added in Microsoft PowerPoint. Gene network map created using https://genemania.org/.

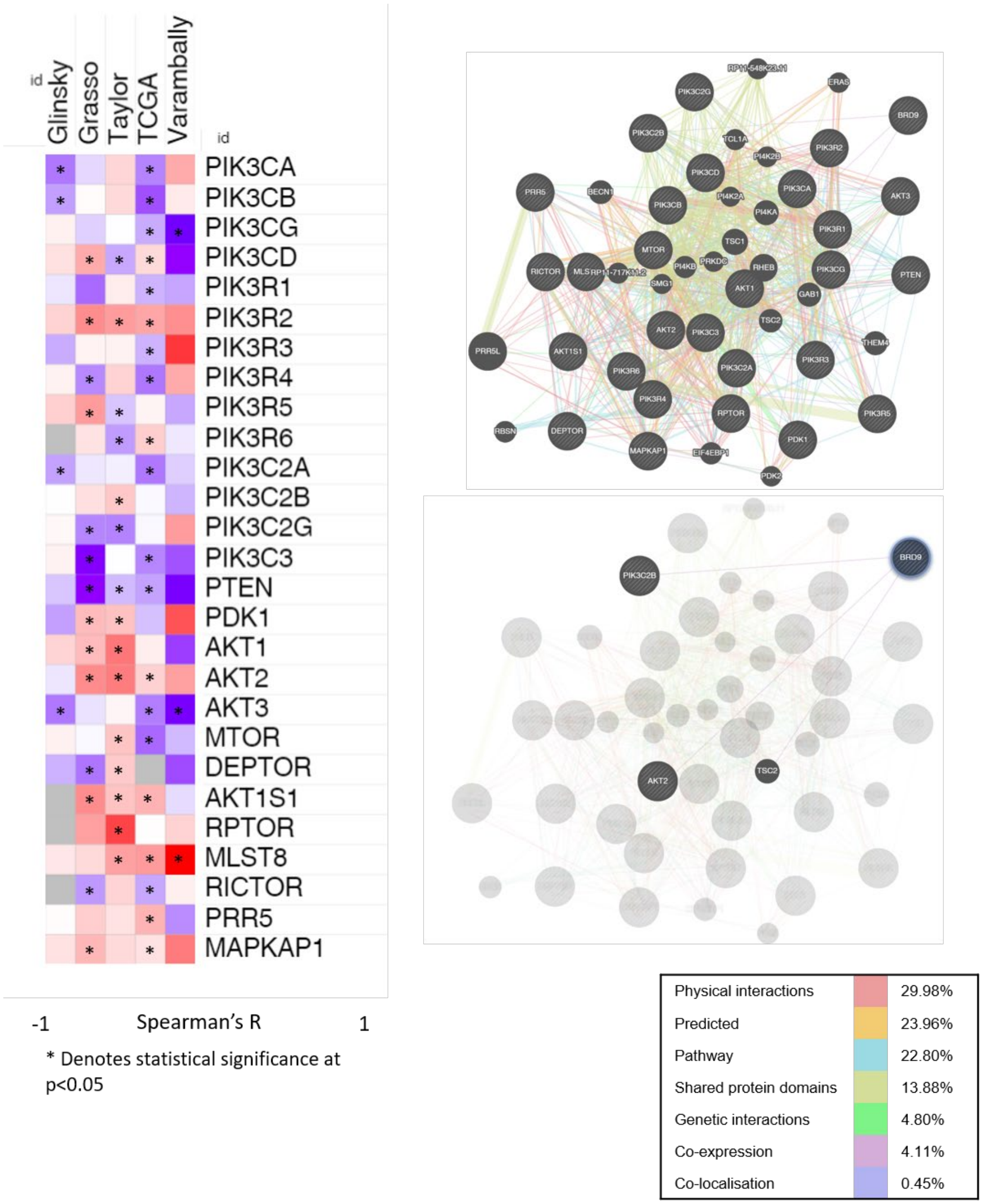

Figure S6. Investigating the correlation and association of *BRD9* with genes expressed in the PI3K-AKT-mTOR pathway. (**A**) Heatmap showing the correlation (Spearman's R coefficient) between *BRD9* and genes involved in the PI3K-AKT-mTOR pathway. Statistically significant correlations (p<0.05) are indicated by '*'. (**B**) Gene network map showing the associations between *BRD9* and known genes expressed in the PI3K-AKT-mTOR pathway. Heatmap made using

https://software.broadinstitute.org/morpheus/ with statistically significant correlation denotation added in Microsoft PowerPoint. Gene network map created using https://genemania.org/.
